# Supplementary material for: A nonmetallic plasmonic catalyst for photothermal CO2 flow conversion with high activity, selectivity and durability
Source: Nat Commun. 2024 Feb 10;15:1273. doi: 10.1038/s41467-024-45516-4 (PMC10858932; doi:10.1038/s41467-024-45516-4)
Supplement: Supplementary file 1 — Supplementary Information [file 41467_2024_45516_MOESM1_ESM.pdf]

Supplementary Information

A nonmetallic plasmonic catalyst for photothermal CO_2 flow conversion with high activity, selectivity and durability

Xueying Wan[1,2,¶], Yifan Li[3,¶], Yihong Chen[1,2,¶], Jun Ma[1,2], Ying-Ao Liu[1,2], En-Dian Zhao[1,2], Yadi Gu[1,2], Yilin Zhao[1,2], Yi Cui[3], Rongtan Li[4], Dong Liu[1,2]*, Ran Long[1], Kim Meow Liew[2,5], and Yujie Xiong[1,2]*

[1]Hefei National Research Center for Physical Sciences at the Microscale, Collaborative Innovative Center of Chemistry for Energy Materials (iChEM), School of Chemistry and Materials Science, National Synchrotron Radiation Laboratory, School of Nuclear Science and Technology, University of Science and Technology of China, Hefei, 230026, Anhui, China.
[2]Suzhou Institute for Advanced Research, Nano Science and Technology Institute, University of Science and Technology of China, Suzhou, 215123, China.
[3]Vacuum Interconnected Nanotech Workstation, Suzhou Institute of Nano-Tech and Nano-Bionics, Chinese Academy of Sciences, Suzhou, 215123, China.
[4]State Key Laboratory of Catalysis, Dalian Institute of Chemical Physics, Chinese Academy of Sciences, Dalian 116023, China
[5]Centre for Nature-Inspired Engineering, Department of Architecture and Civil Engineering, City University of Hong Kong, Kowloon, Hong Kong, China.
[¶]These authors contributed equally: Xueying Wan, Yifan Li, Yihong Chen.

*email: dongliu@ustc.edu.cn; yjxiong@ustc.edu.cn

This file includes:

Supplementary text
Supplementary Figs 1 to 22
Supplementary Tables 1 to 4
Supplementary references 1 to 25

Experimental section

Materials

Mo powder was purchased from Macklin Ltd. (Shanghai, China). Hydrogen peroxide and ethanol were produced from Shanghai Chemical Reagent Factory. The commercial MoO_2, Mo_2N and MoN were purchased from Boer, Energy Chemical and RHAWN Co. Ltd. (Shanghai, China), respectively. The purity quotients of Ar, NH_3, CO_2 and H_2 were 99.999%. Deionized water was prepared from an ultrapure water system.

Calculation of energy conversion efficiency

Light energy to chemical energy conversion efficiency (LTC) calculation of photothermal RWGS reaction.[1] The LTC of photothermal RWGS demonstration was calculated as follows:

$$\mathrm{LTC} = \frac{R_{CO} \times \Delta G^{548\ K}}{P_{lamp} \times S} \times 100\%$$

where R_{co} is the product production rate (mmol·s^{-1}), $\Delta G^{573\ K}$ is the Gibbs free energy at 573 K of RWGS reaction (CO_2 (g) + H_2 (g) = CO (g) + H_2O (g), $\Delta G^{573\ K}$ = 19.52 kJ·mol^{-1}), P_{lamp} is the light intensity (3 W·cm^{-2}), and S is the illumination area of catalyst (0.785 cm^2, a disc with the diameter of 1 cm). ΔG was calculated by HSCChemistry at different temperatures.

Input energy to chemical energy conversion efficiency (ITC) calculation of thermal RWGS reaction. [2] Due to the thermal catalytic reaction occurring in the laboratory, the power of the reaction device was used as the input energy. The heat loss

was temporarily not considered in this system. The ITC of thermal RWGS demonstration was calculated as follows:

$$\text{ITC} = \frac{\text{R}_{\text{CO}} \times \Delta\text{G}}{\text{P}_{\text{device}}} \times 100\%$$

where ΔG is the Gibbs free energy of RWGS reaction, and P_{device} is the power consumption of reaction devices (1.5 kW).

Thermal energy to chemical energy conversion efficiency (TTC) calculation of thermal RWGS reaction.[2] Assuming under ideal conditions, thermal energy was used to heat the reactants from room temperature to the target temperature in the adiabatic reactor. The TTC of thermal RWGS demonstration was calculated as follows:

$$\text{TTC} = \frac{\text{R}_{\text{CO}} \times \Delta\text{G}}{\sum_{\text{i}} \text{C}_{\text{i}}\text{m}_{\text{i}}\Delta\text{T}} \times 100\%$$

where C_i is the specific heat capacity of reactant (C_{H2} = 14.3 kJ·kg^{-1}·K^{-1} and C_{CO2} = 1.0 kJ·kg^{-1}·K^{-1}), m_i is the gas flow rate (kg·s^{-1}), and ΔT is the temperature difference from room temperature to target temperature.

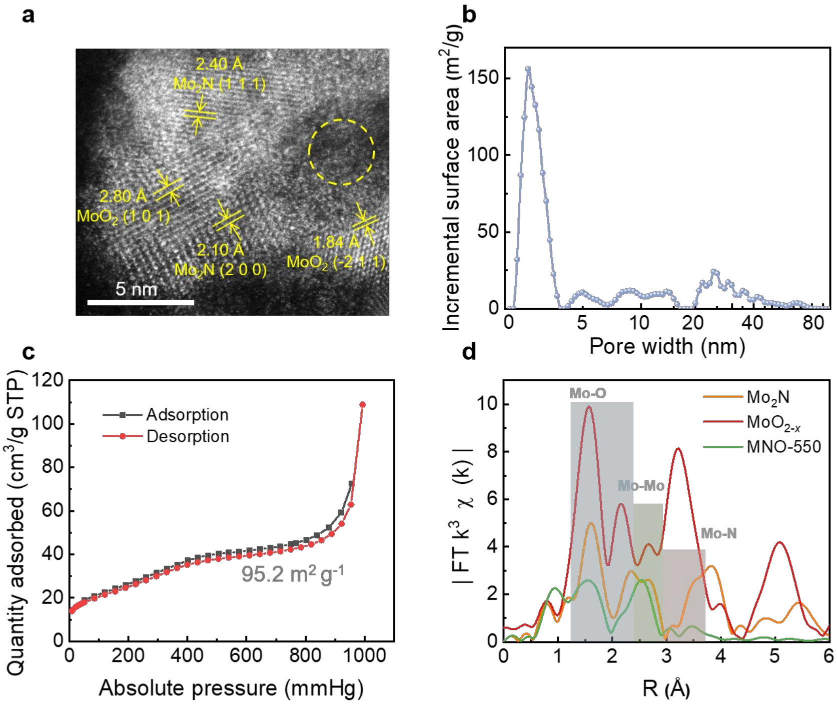

Supplementary Fig. 1 | The structural characterizations of catalyst. **a**, Atomic-level HAADF-STEM image, **b**, pore size distribution and **c**, N_2 adsorption-desorption isotherms of MNO-550. **d**, k^3-weighted Mo K-edge FT-EXAFS spectra of the MNO-550 in reference to Mo_2N and MoO_{2-x}.

HAADF-STEM image reveals a clearer structure of Mo_2N/MoO_{2-x} nanosheets. The lattices of Mo_2N and MoO_2 as well as the pore structure are observed more accurately (Supplementary Fig. 1a). Mo_2N/MoO_{2-x} nanosheets have a high specific surface area of 95.2 $m^2 \cdot g^{-1}$ with a pore width of ~3 nm (Supplementary Fig. 1b and 1c), which is consistent with HRTEM result. Moreover, the Mo K-edge of FT-EXAFS spectra indicates the coexistence of Mo_2N and MoO_2 in MNO-550 (Supplementary Fig. 1d).[3,4]

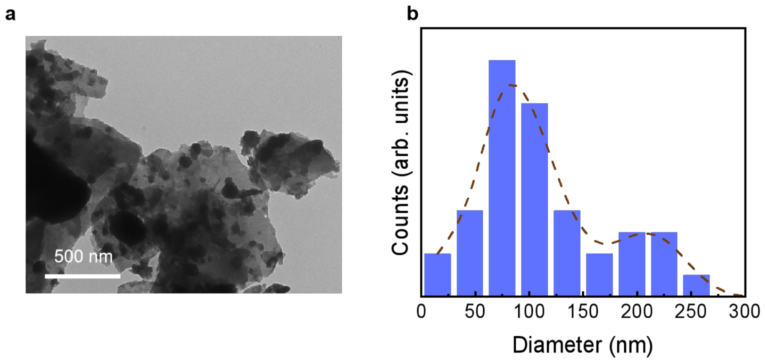

Supplementary. Fig. 2 | The morphology characterizations of catalyst. **a**, TEM image and **b**, grain size distribution of Mo_2N/MoO_{2-x} nanosheets.

The plasmon resonance is determined by the size, shape and material of the nanoparticle. The size distribution of Mo_2N/MoO_{2-x} nanosheets is from 20 nm to 250 nm, which belongs to a wide range. The plasmon peaks of Mo_2N/MoO_{2-x} nanosheets with different grain sizes would overlap and cover each other, so that the maximum absorption peak cannot be displayed significantly (Fig. 2a).[5,6]

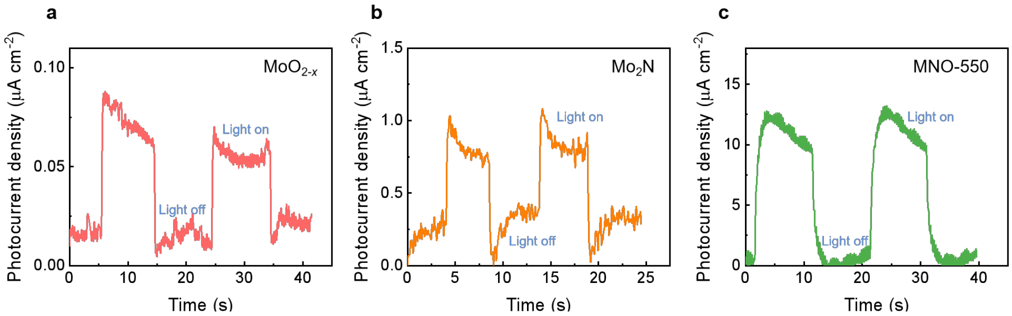

Supplementary. Fig. 3 | The photocurrent response of catalyst. **a-c**, Photocurrent response of (**a**) MoO_{2-x}, (**b**) Mo_2N and (**c**) MNO-550 under full-spectrum illumination.

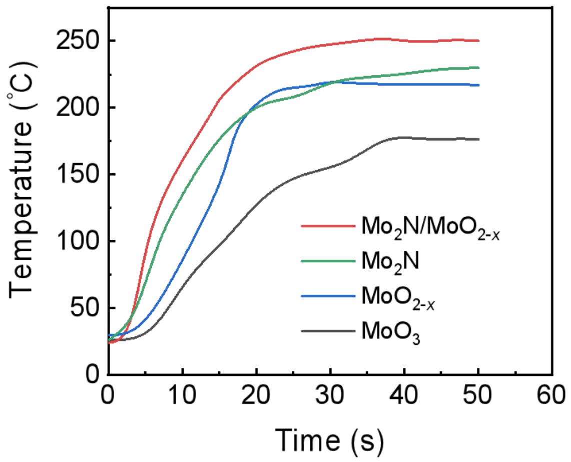

Supplementary Fig. 4 | The surface temperature profiles of catalyst. Surface temperature profiles of Mo_2N/MoO_{2-x}, Mo_2N, MoO_3 and MoO_{2-x} catalysts under the illumination of 3.0 W·cm^{-2}.

We measure the surface temperature profiles of Mo_2N/MoO_{2-x}, Mo_2N, MoO_3 and MoO_{2-x} under illumination of 3.0 W·cm^{-2}. Due to the LSPR effect, Mo_2N/MoO_{2-x} can quickly reach up to 250 °C within 25 seconds (Supplementary Fig. 4).

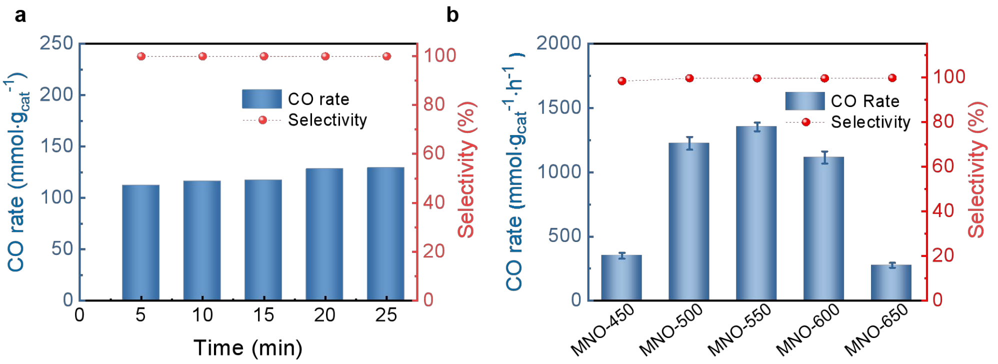

Supplementary Fig. 5 | The time- and catalyst-dependent RWGS performance. The production rate and selectivity of CO evolution for photothermal catalytic RWGS reaction in a batch reactor **a**, by MNO-550 at different reaction times and **b**, of Mo_2N/MoO_{2-x} with different annealing temperature under 3 $W \cdot cm^{-2}$ light illumination for 5 min.

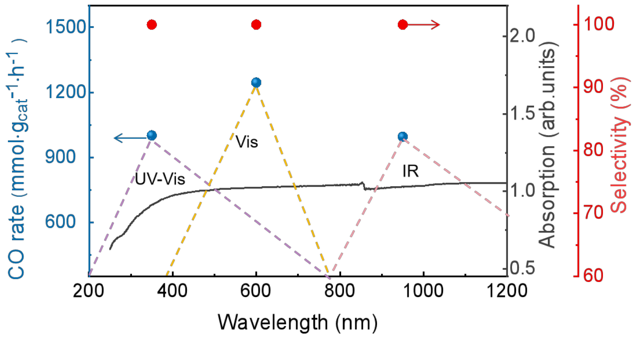

Supplementary Fig. 6 | The relevance between RWGS performance and light wavelength. The CO yield rate and selectivity of MNO-550 under light illumination with different wavebands for 5 min.

We have used different wavebands of light to stimulate reactions to comprehend the function of light (a light density of 2 W·cm^{-2}). The CO yield rate of MNO-550 positively correlates with the absorption spectra. Due to the superimposed plasmonic characters of MoO_{2-x} and Mo_2N in the visible light region, the CO yield rate can reach up to 1180 mmol·g_{cat}^{-1}·h^{-1} for 5 min under visible light excitation.

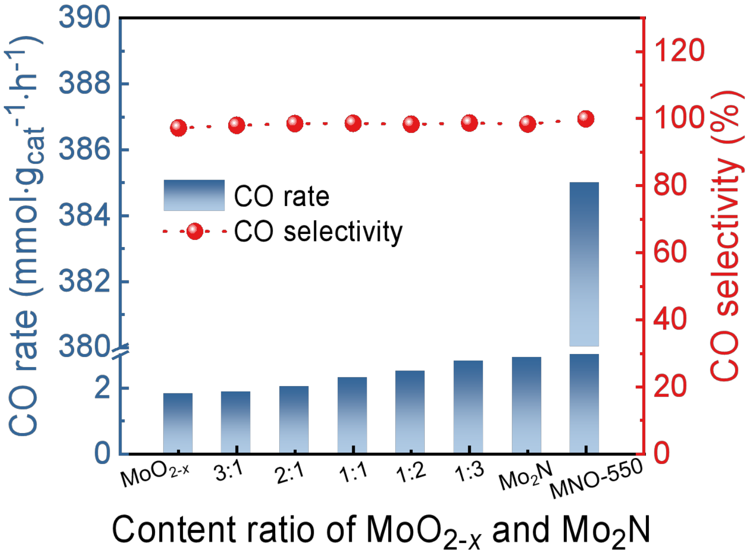

Supplementary Fig. 7 | The RWGS performance of physically mixed catalysts. The production rate and selectivity of CO evolution for physically mixed Mo_2N and MoO_{2-x} catalysts under 3 W·cm^{-2} full-spectrum light irradiation. Data of MNO-550 are also listed for comparison.

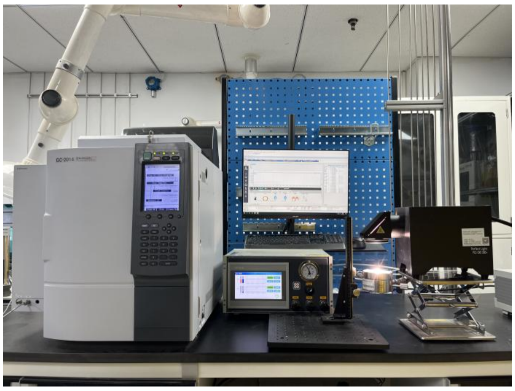

Supplementary Fig. 8 | Photograph of the flow reactor testing device.

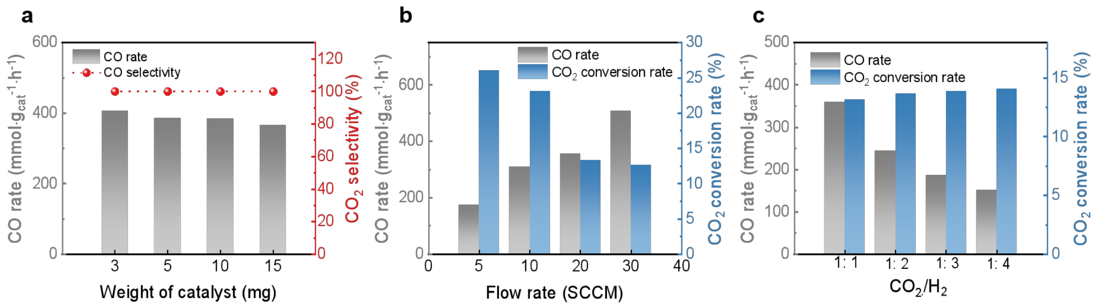

Supplementary Fig. 9 | The RWGS performance under different conditions. **a**, The production rate and selectivity of MNO-550 for photothermal catalytic RWGS reaction at different dosages of catalyst. **b**, The CO production rate and CO_2 conversion rate of MNO-550 in various flow rates (CO_2/H_2 1:1) for photothermal catalytic RWGS reaction. **c**, The CO production rate and CO_2 conversion rate of MNO-550 with different proportions of mixed gas under the flow rate of 20 SCCM for photothermal catalytic RWGS reaction.

We have investigated the photothermal catalytic reaction performance of MNO-550 under different conditions. The catalyst weight has little effect on the product generation rate (Supplementary Fig. 9a). As the flow rate of the reaction gases increases, the production rate is promoted while the CO_2 conversion rate is reduced accordingly. When the proportion of H_2 in the mixed gas increases, the CO_2 conversion rate is slightly promoted but the CO production rate is reduced.

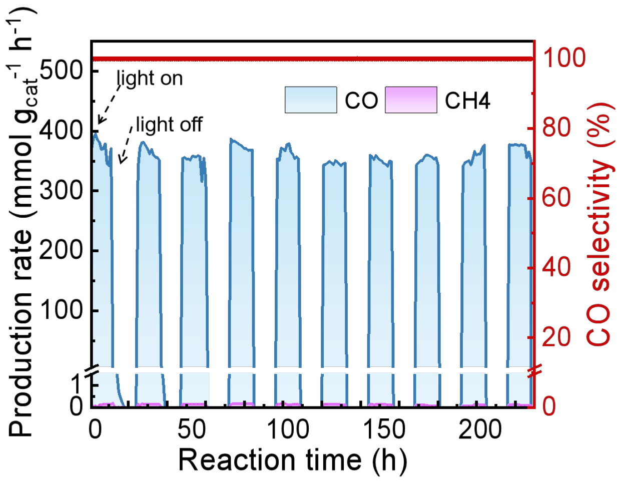

Supplementary Fig. 10 | The stability of catalyst during repetitive on/off experiments. The CO generation rate and selectivity of MNO-550 under long-term alternating on/off light conditions (3W·cm^{-2}).

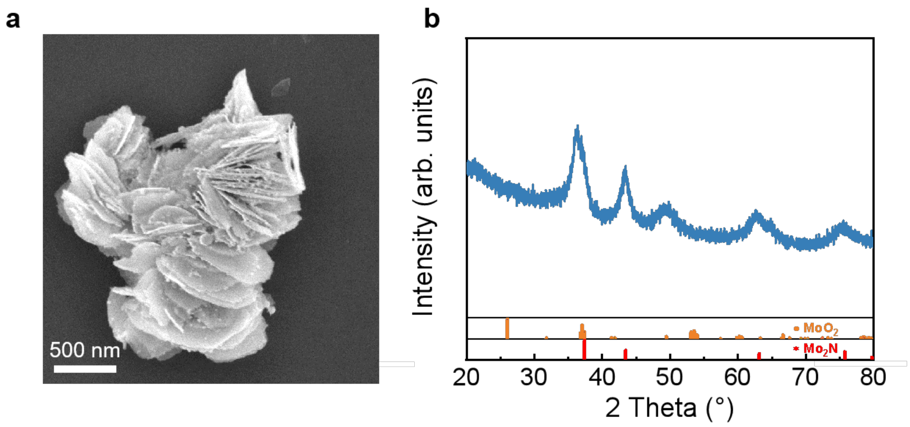

Supplementary Fig. 11 | The morphology characterization of catalyst after reaction. **a**, SEM image and **b**, XRD pattern of MNO-550 after photothermal catalytic reaction.

Supplementary Fig.11 shows the SEM image and XRD pattern of MNO-550 after photothermal catalytic reaction. The morphology and phase remain almost unchanged, which indicates that the catalyst has good photothermal stability.

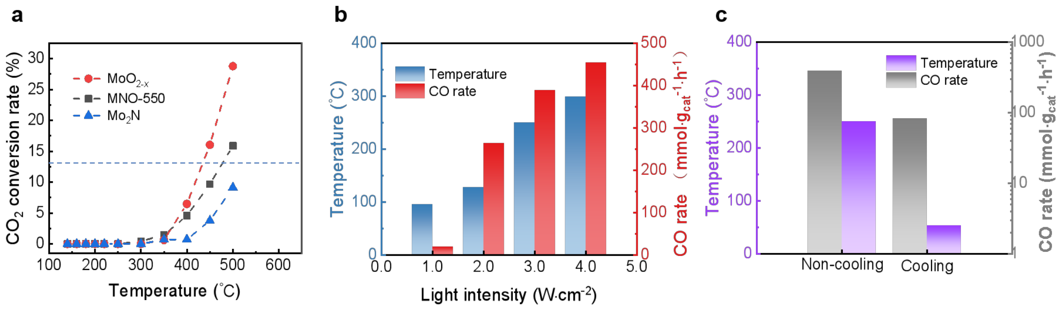

Supplementary Fig. 12 | The effects of heat on the RWGS performance. **a**, The CO_2 conversion rate and CO selectivity of MNO-550, commercial Mo_2N and MoO_{2-x} at various temperatures in thermal catalysis. **b**,**c**, The surface temperatures and CO generation rates of MNO-550 under (**b**) different light intensities and (**c**) different cooling environment with 3.0 $W\cdot cm^{-2}$ light intensity.

We evaluate the thermal catalytic performance of MNO-550, commercial Mo_2N and MoO_{2-x}. When the CO_2 conversion rate reaches up to 13.1%, the reaction temperatures need to be above 420 °C (Supplementary Fig. 12a). The practical reaction temperature is usually higher than 400 °C in thermal catalysis.[7,8] However, the corresponding temperature in photothermal catalysis is only 250 °C, suggesting that the synergy effect of photon and thermal energy can improve reaction activity. As shown in Supplementary Fig. 12b, the surface temperature and CO generation rate are promoted with increasing light intensities. When we use ice to cool down the temperature of the surrounding environment, both the surface temperature of the sample and the generation rate of CO decrease (Supplementary Fig. 12c). Nevertheless, the photocatalytic RWGS performance of the cooling sample is still much higher than that of thermal catalysis at 250 °C (equal to the photothermal temperature generated by LSPR) without light illumination.[9]

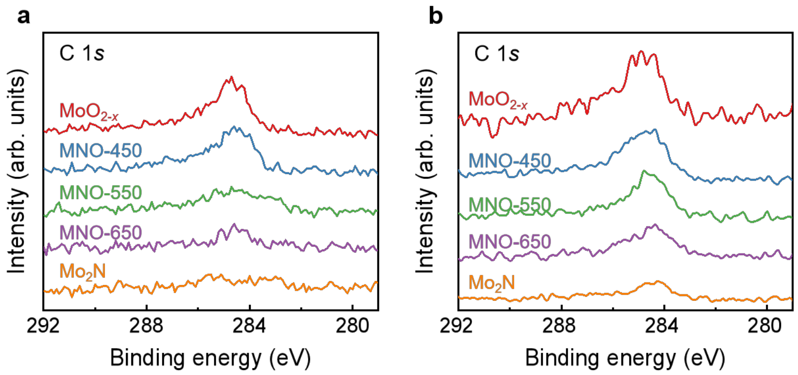

Supplementary Fig. 13 | In-situ XPS C 1*s* spectra. **a**,**b**, XPS spectra of C 1*s* for different samples (a) before and (b) after the reaction.

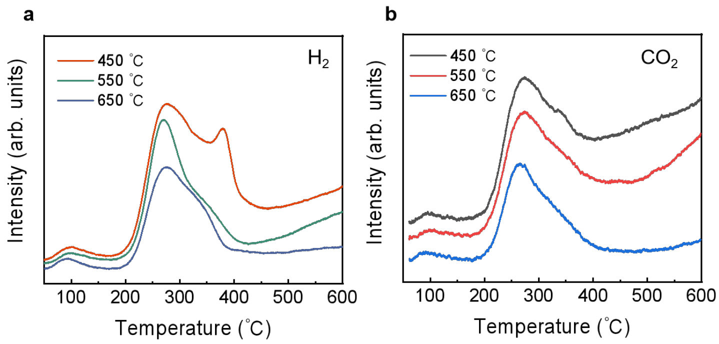

Supplementary Fig. 14 | H_2 and CO_2-TPD spectra. **a**, H_2-TPD and **b**, CO_2-TPD results of Mo_2N/MoO_{2-x} samples prepared with different annealing temperatures.

The ratio of N atom and oxygen vacancy can be adjusted by changing the annealing temperature, which affects the adsorption of reaction gases and the activity of catalysts. MNO-550 shows moderate adsorption capacity for CO_2 and H_2 among the Mo_2N/MoO_{2-x} samples. Thus, MNO-550 achieves the best synergy component and the optimal activity at an annealing temperature of 550 °C for the suitable ratio of N atom to O vacancy.

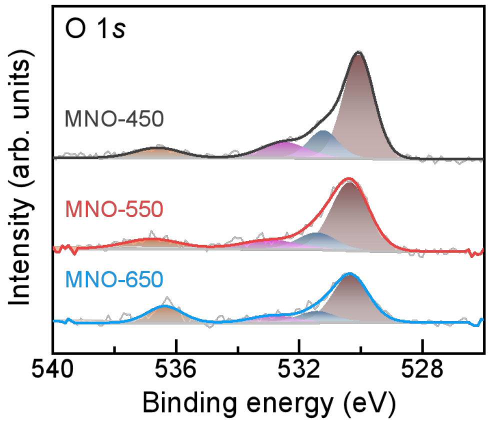

Supplementary Fig. 15 | In-situ XPS O 1*s* spectra. In-situ O 1*s* NAP-XPS spectra for Mo_2N/MoO_{2-x} samples in 0.3 mbar CO_2 at room temperature without illumination. The magenta, blue and brown peaks are associated with oxygen originating from adsorbed CO_2, oxygen vacancy and metal oxide, respectively.

It can be evidently observed that the peak intensity of adsorbed CO_2, oxygen vacancy and metal oxide decrease all monotonously with increasing annealing temperatures. MNO-550 exhibits a moderate adsorption capacity among three Mo_2N/MoO_{2-x} samples.

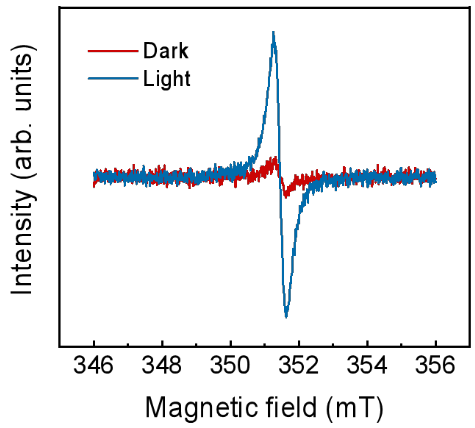

Supplementary Fig. 16 | In-situ EPR spectra. In-situ EPR spectra for Mo_2N/MoO_{2-x} in the dark and under full-spectrum light irradiation.

The electron transfer behavior in Mo_2N/MoO_{2-x} can be further confirmed by in-situ EPR measurement. The signal increases significantly upon light irradiation, indicating the creation of oxygen vacancies by plasmonic hot electrons in the catalyst.

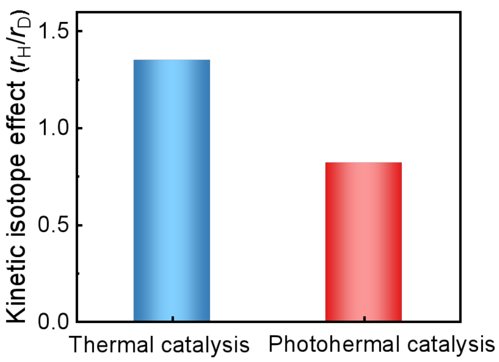

Supplementary Fig. 17 | The KIE results for thermal catalysis and photothermal catalysis.

The reaction rate decreases in thermal catalysis when the H_2 is replaced by D_2 (KIE = 1.35). However, the KIE value of Mo_2N/MoO_{2-x} in photothermal catalysis is 0.82 (Fig. R2/Supplementary Fig. 16). The apparent change of KIE value reflects the transformation of hydrogen-involved behavior under different driving conditions for RWGS reaction, which proves the irreplaceable role of photothermal catalysis to promote H_2 activation.

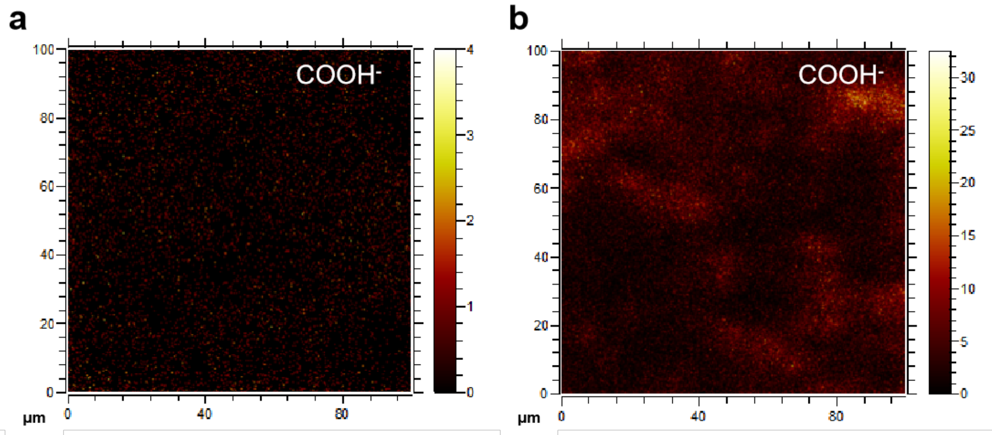

Supplementary Fig. 18 | The TOF-SIMS profiles of *$COOH^-$. a,b, TOF-SIMS profiles of *$COOH^-$ (m/z=45.02) for MNO-550 catalyst (**a**) before and (**b**) after the reaction.

We can observe that the MNO-550 after reaction exhibits the markedly increased signals of carbon-containing intermediates, *i.e.*, *$CO_2^{\delta-}$ and *$COOH^-$. In particular, the sharply increased *$COOH^-$ signal coincides well with the NAP-XPS results.

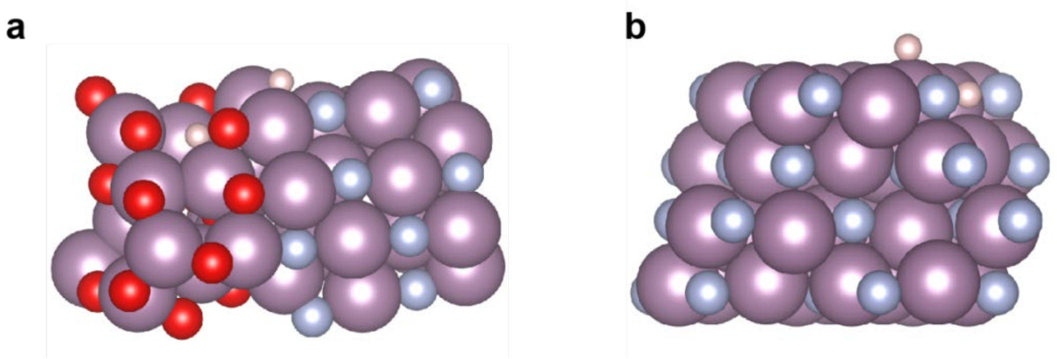

Supplementary Fig. 19 | The configurations of H_2 dissociative adsorption. **a**,**b**, Configurations of H_2 dissociative adsorption at the surface of (**a**) Mo_2N/MoO_{2-x} and (**b**) Mo_2N.

As shown in Supplementary Fig. 18, H_2 molecule prefers dissociative adsorption at the surface of Mo_2N/MoO_{2-x} and Mo_2N in the form of two independent hydrogen atoms. As for MoO_2, H_2 molecules are directly adsorbed on the surface as shown in Fig. 4a.

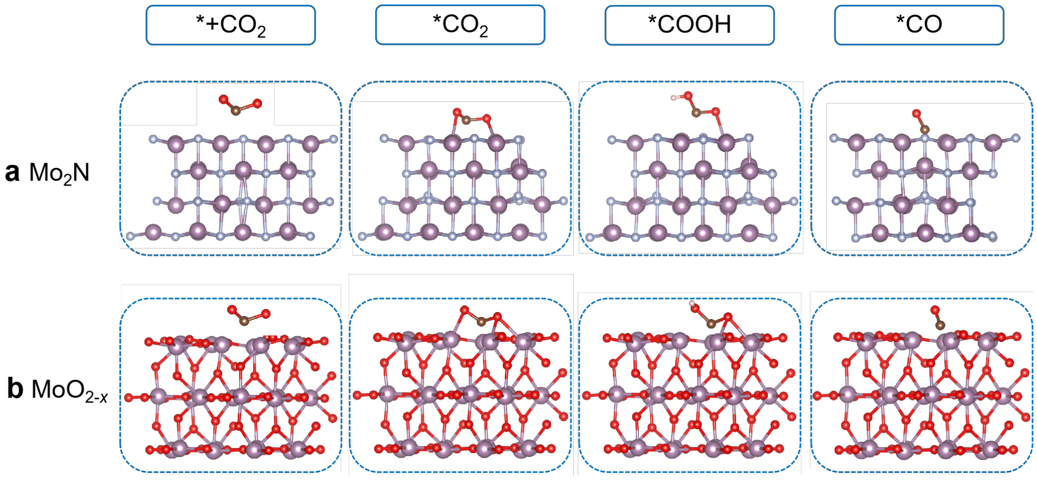

Supplementary Fig. 20 | The configurations of CO_2 reduction to CO. **a**,**b**, Configurations of CO_2 reduction to CO at the surface of (**a**) Mo_2N and (**b**) MoO_{2-x}.

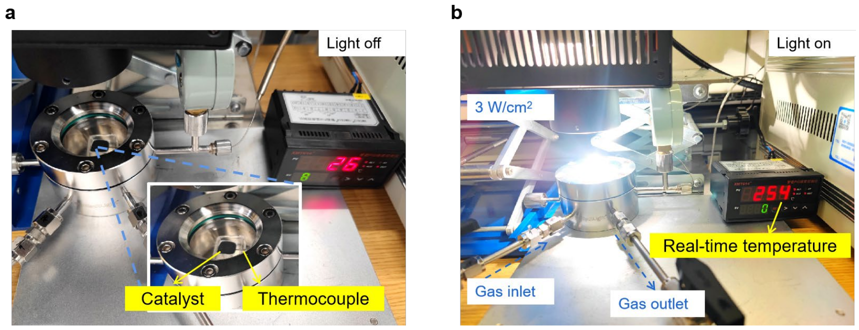

Supplementary Fig. 21 | Schematic diagram of in-situ temperature measurement for reaction device. **a**,**b**, The real-time photos of the reaction device and corresponding measured temperatures (**a**) before and (**b**) during light illumination.

We use copper foil with high thermal conductivity as the substrate to load sample for ensuring the accuracy and reliability of measured temperature. As shown in the Supplementary Fig. 22 below, when the catalyst is not loaded, the real-time temperature rise curves of Cu foil without catalyst and thermocouple directly irradiated by the light source are basically identical (~70 °C). It proves the reliability of this method. However, when loading the catalyst, the real-time sample temperature can quickly rise to 250 °C.

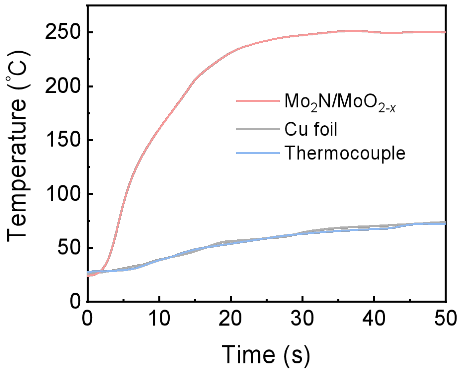

Supplementary Fig. 22 | The temperature rise curves. The corresponding temperature rise curves of catalyst on Cu foil, pure Cu foil and only thermocouple.

Supplementary Table 1. The ratio of Mo_2N to MoO_2 calculated by peak fitting of Mo 3p.

Sample	MoO_{2-x}	MNO-450	MNO-550	MNO-650	Mo_2N
ratio of Mo_2N to MoO_2	0	0.22	0.60	0.68	0.80

Supplementary Table 2. Calculated energy conversion efficiency of MNO-550 in photothermal catalysis under 3 W·cm^{-2} full-spectrum illumination and in thermal catalysis at various reaction temperatures.

	Flow rate CO_2/H_2 1:1	Reaction temperature (℃)	CO_2 conversion rate (%)	ΔG (kJ·mol^{-1})	CO yield (mmol·s^{-1})	TTC (%)	LTC (%)
Photothermal catalysis	20 SCCM	250*	13.1	19.52	9.86×10^{-4}	15.84	0.82
	10 SCCM	250*	23.0	19.52	8.58×10^{-4}	27.60	0.71
	**Flow rate CO_2/H_2 1:1**	**Reaction temperature**	**CO_2 conversion rate (%)**	**ΔG (kJ·mol^{-1})**	**CO yield (mmol·s^{-1})**	**TTC (%)**	**ITC (%)**
Thermal catalysis	9.6 SCCM	250	0.0	19.52	non-detected	0.0	0.0
	9.6 SCCM	300	0.407	17.61	1.45×10^{-5}	0.36	0.000017
	9.6 SCCM	485	13.1	10.89	4.68×10^{-4}	4.27	0.00034
	9.6 SCCM	500	15.9	10.36	5.68×10^{-4}	4.78	0.00039
	9.6 SCCM	550	22.7	8.39	8.11×10^{-4}	5.00	0.00045

*LSPR-induced photothermal temperature, without external heat supply.

As shown in Table 2, RWGS reaction does not occur at 250 °C through thermal catalysis in this system. Photothermal catalysis can lower the requirement for reaction temperature effectively. To achieve a similar CO_2 conversion rate, the thermal energy conversion efficiency of MNO-550 in photothermal catalysis is 4~5 times higher than that in thermal catalysis, while the required reaction temperature can be reduced by 230~300 °C. Comparing the LTC and ITC, we can find that the energy consumption of photothermal catalysis is substantially lower than that of thermal catalysis, which reflects the advantages of this plasmonic photothermal catalysis in catalytic performance as well as thermal management in contrast with thermal catalysis. When the reaction temperature hits 550 °C in thermal catalysis, the CO_2 conversion rate can reach up to 23%. For photothermal catalytic RWGS reaction, it only needs a mild

reaction condition (3 W·cm^{-2} full-spectrum illumination without external heat supply) to achieve the similar performance.

Supplementary Table 3. The specific testing conditions and the photothermal activities of Mo_2N/MoO_{2-x} and state-of-the-art catalysts.

Catalyst	Light intensity	External heating temperature	CO_2/H_2 ratio	CO yield rate ($mmol \cdot g_{cat}^{-1} \cdot h^{-1}$)	Long-term stability test	Activity decay after stability test
Mo_2N/MoO_{2-x} (**This work**)	3 $W \cdot cm^{-2}$	/	1:1	1345 (reaction for 5min)	/	/
	1 $W \cdot cm^{-2}$	/	1:1	18.1	/	/
	2 $W \cdot cm^{-2}$	/	1:1	264	/	/
	3 $W \cdot cm^{-2}$	/	1:1	355	190	~12%
	4 $W \cdot cm^{-2}$	/	1:1	454	/	/
Ni_3N [10]	3.06 $W \cdot cm^{-2}$	/	20:1	1212	/	/
	2.5 $W \cdot cm^{-2}$	/	20:1	325	25	~30%
Ru/Mo_2TiC_2 [11]	3.4 $W \cdot cm^{-2}$	/	1:1	312	15	~3%
Au/TiO_2 [12]	1.4 $W \cdot cm^{-2}$	/	1:1	13.4	4	/
Au/TiO_2 (DP) [13]	0.52 $W \cdot cm^{-2}$	400 ℃	1:2	159.8	/	/
$Ga\text{-}Cu/CeO_2$ [14]	3.82 $W \cdot cm^{-2}$	/	1:1	337.2	10	~28%
CF-Cu_2O [15]	4 $W \cdot cm^{-2}$	/	5:1	139.6	60	~13 %
$Ni@SiO_2$ [16]	2.8 $W \cdot cm^{-2}$	/	1:1	44.1	35	~2%
Cu/2D-Si [17]	3.4 $W \cdot cm^{-2}$	/	1:4	13	5	/
$Pd@Nb_2O_5$ [18]	4.2 $W \cdot cm^{-2}$	/	1:1	18.8	3.5	/
$Ni_{12}P_5/SiO_2$ [19]	0.8 $W \cdot cm^{-2}$	/	5:1	13.5	100	/
	2.3 $W \cdot cm^{-2}$	/	5:1	960	/	/
$TiN@TiO_2@In_2O_{3-x}(OH)_y$ [20]	1.6 $W \cdot cm^{-2}$	300 ℃	1:3	13.04	40	~10%
Fe_3O_4 [21]	2.05 $W \cdot cm^{-2}$	/	1:2	11.3	24	/
Mo_2NH_x [22]	0.35 $W \cdot cm^{-2}$	175 ℃	1:4	0.35	15	/
Black In_2O_3 [23]	2..0 $W \cdot cm^{-2}$	/	1:1	1.87	70	~6%
$CuNi/CeO_2$ [24]	Visible light	310 ℃	1:4	1.3	25	/
Pt/H_xMoO_{3-y} [25]	Visible-infrared	140 ℃	1:1	1.2	6	/

* "~" refers to the approximation for each reference according to their provided mass and reaction time of catalysts in the experimental section.

Supplementary Table 4. Calculated free energies (eV) of Mo_2N/MoO_{2-x}, Mo_2N and MoO_{2-x} in CO_2 reduction to CO.

Species	Mo_2N/MoO_{2-x}	Mo_2N	MoO_{2-x}
$*CO_2$	-1.71 eV	-1.31 eV	-2.52 eV
*COOH	-1.80 eV	-1.16 eV	-2.14 eV
*CO	-4.12 eV	-0.62 eV	-1.79 eV

Supplementary References

1 Li, Y. *et al.* Cu-based high-entropy two-dimensional oxide as stable and active photothermal catalyst. *Nat. Commun.* **14**, 3171 (2023).

2 Lou, X. *et al.* Highly efficient photothermal catalytic upcycling of polyethylene terephthalate via boosted localized heating. *Chinese J. Catal.* **49**, 113-122 (2023).

3 Chen, W. *et al.* Rational design of single molybdenum atoms anchored on N-doped carbon for effective hydrogen evolution reaction. *Angew. Chem. Int. Ed.* **56**, 16086-16090 (2017).

4 Liu, Z. *et al.* EXAFS study of g-Mo_2N and Mo nitrides supported on zeolites. *Mater. Lett.* **54**, 364-371 (2002).

5 Rej, S. *et al.* Colloidal titanium nitride nanobars for broadband inexpensive plasmonics and photochemistry from visible to mid-IR wavelengths. *Nano Energy* **104**, 107989 (2022).

6 Chang, C. *et al.* Highly plasmonic titanium nitride by room-temperature sputtering. *Sci. Rep.* **9**, 15287 (2019).

7 Xin, H. *et al.* Overturning CO_2 hydrogenation selectivity with high activity via reaction-induced strong metal-support interactions. *J. Am. Chem. Soc.* **144**, 4874-4882 (2022).

8 Lin, L. *et al.* Reversing sintering effect of Ni particles on gamma-Mo_2N via strong metal support interaction. *Nat. Commun.* **12**, 6978 (2021).

9 Shao, T. *et al.* A stacked plasmonic metamaterial with strong localized electric field enables highly efficient broadband light-driven CO_2 hydrogenation. *Adv. Mater.* **34**, 2202367 (2022).

10 Singh, S. *et al.* Surface plasmon-enhanced photo-driven CO_2 hydrogenation by hydroxy-terminated nickel nitride nanosheets. *Nat. Commun.* **14**, 2551 (2023).

11 Wu, Z. *et al.* Mo_2TiC_2 MXene-supported Ru clusters for efficient photothermal reverse water-gas shift. *ACS Nano* **17**, 1550-1559 (2023).

12 Molina, P. M. *et al.* Low temperature sunlight-powered reduction of CO_2 to CO using a plasmonic Au/TiO_2 nanocatalyst. *Chemcatchem* **13**, 4507-4513 (2021).

13 Upadhye, A. A. *et al.* Plasmon-enhanced reverse water gas shift reaction over oxide supported Au catalysts. *Catalysis Science & Technology* **5**, 2590-2601 (2015).

14 Deng, B., Song, H., Peng, K., Li, Q. & Ye, J. Metal-organic framework-derived Ga-Cu/CeO_2 catalyst for highly efficient photothermal catalytic CO_2 reduction. *Appl. Catal. B* **298**, 120519 (2021).

15 Wan, L. *et al.* Cu_2O nanocubes with mixed oxidation-state facets for (photo)catalytic hydrogenation of carbon dioxide. *Nat. Catal.* **2**, 889-898 (2019).

16 Wang, S. *et al.* Grave-to-cradle upcycling of Ni from electroplating wastewater to photothermal CO_2 catalysis. *Nat. Commun.* **13**, 5305 (2022).

17 Su, Y. *et al.* High surface area siloxene for photothermal and electrochemical catalysis. *Nanoscale* **15**, 154-161 (2022).

18 Jia, J. *et al.* Visible and near-infrared photothermal catalyzed hydrogenation of gaseous CO_2 over nanostructured Pd@Nb_2O_5. *Adv. Sci.* **3**, 2198-3844 (2016).

19 Xu, Y. *et al.* High-performance light-driven heterogeneous CO_2 catalysis with near-unity selectivity on metal phosphides. *Nat. Commun.* **11**, 5149 (2020).
20 Nguyen, N. T. *et al.* Plasmonic titanium nitride facilitates indium oxide CO_2 photocatalysis. *Small* **16**, 2005754 (2020).
21 Song, C. *et al.* Photothermal conversion of CO_2 with tunable selectivity using Fe-based catalysts: From oxide to carbide. *ACS Catal.* **10**, 10364-10374 (2020).
22 Sun, M. *et al.* Photoinduced H_2 heterolysis to form Mo_2NH_x active species for CO_2 reduction. *ACS Energy Lett.* **6**, 2024-2029 (2021).
23 Wang, L. *et al.* Black indium oxide a photothermal CO_2 hydrogenation catalyst. *Nat. Commun.* **11**, 2432 (2020).
24 Yue, X. *et al.* Visible light-regulated thermal catalytic selectivity induced by nonthermal effects over CuNi/CeO_2. *Chem. Eng. J.* **458**, 141491 (2023).
25 Ge, H., Kuwahara, Y., Kusu, K. & Yamashita, H. Plasmon-induced catalytic CO_2 hydrogenation by a nano-sheet Pt/H_xMoO_{3-y} hybrid with abundant surface oxygen vacancies. *J. Mater. Chem. A* **9**, 13898-13907 (2021).
